# Technoeconomic and environmental analysis of cryogenic and MQL-assisted machining of Hastelloy X

**DOI:** 10.1038/s41598-025-07526-0

**Published:** 2025-07-01

**Authors:** Binayak Sen, V. V. Murali Krishnam Raju, Raman Kumar, T. Ramachandran, Ashwin Jacob, Jajneswar Nanda, Gurbhej Singh, Abhijit Bhowmik, Jibitesh Kumar Panda

**Affiliations:** 1 Centre for Computational Modeling, Chennai Institute of Technology, Chennai, 600069 Tamil Nadu India; 2 Department of Mechanical Engineering, Chennai Institute of Technology, Chennai, 600069 Tamil Nadu India; 3 Department of Mechanical Engineering, SRKR Engineering College, Bhimavaram, 534204 Andhrapradesh India; 4 University School of Mechanical Engineering, Rayat Bahra University, Kharar, 140103 Punjab India; 5 Faculty of Engineering, Sohar University, PO Box 44, Sohar, PCI 311 Oman; 6 Department of Mechanical Engineering, School of Engineering and Technology, JAIN (Deemed to be University), Bangalore, Karnataka India; 7 Department of Mechanical Engineering, Sathyabama Institute of Science and Technology, Chennai, Tamil Nadu India; 8 Department of Mechanical Engineering, Siksha ’O’ Anusandhan (Deemed to be University), Bhubaneswar, 751030 Odisha India; 9 Department of Mechanical Engineering, Amritsar Group of Colleges, Amritsar, 143109 India; 10 Department of Additive Manufacturing, Mechanical Engineering, SIMATS, Saveetha Institute of Medical and Technical Sciences, Thandalam, Chennai, 602105 India; 11 Divison of Research and Development, Lovely Professional University, Punjab, Phagwara India; 12 Department of Mechatronics, Manipal Institute of Technology, Manipal Academy of Higher Education, Manipal, Karnataka India

**Keywords:** Hastelloy X, Machining responses, Energy consumption, Machining cost, Sustainable manufacturing, Biochemistry, Environmental sciences

## Abstract

The growing significance of superalloys like Hastelloy X, particularly in critical engineering sectors such as aerospace, chemical processing, and selective biomedical equipment (e.g., surgical instruments and medical tooling), underscores the need for advancements in their manufacturing processes. In today’s era of advanced manufacturing, it is crucial to develop machining systems that are both environmentally sustainable and cost-effective. To bridge the existing gap between economic, technological, and sustainability aspects in the machining of Hastelloy X, the present research aims to shed light on this critical interplay. Experimental investigations were conducted to evaluate the performance of various cooling techniques, including dry machining, minimum quantity lubrication (MQL), and cryogenic cooling using liquid nitrogen (LN₂) and carbon dioxide (CO₂). The results revealed that cryogenic cooling with LN₂ demonstrated superior performance across technological, sustainability, and economic metrics, outperforming other methods. Specifically, LN₂ cooling during the turning of Hastelloy X led to a reduction in tool wear and surface roughness by 21.11% and 25%, respectively, over dry machining conditions. These findings highlight the potential of advanced lubrication and cooling techniques to enhance sustainable manufacturing practices, reducing resource consumption while improving machining performance, particularly for industries involving difficult-to-machine superalloys.

## Introduction

**Fig. 1:**
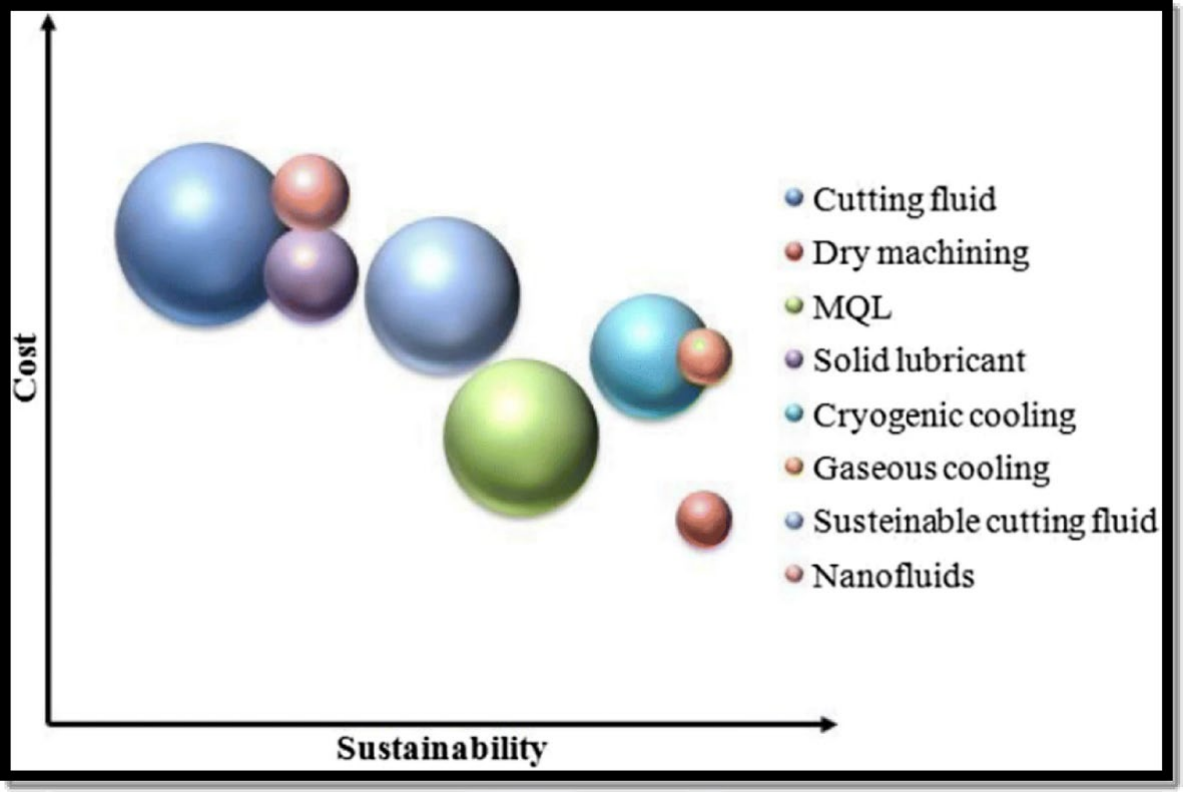
Comparison of lubrication/cooling mediums based on cost and sustainability metrics^11^.

Nickel-based superalloys are essential in the manufacturing of critical components requiring exceptional performance, including superior resistance to oxidation, creep, and corrosion, as well as high strength, making them ideal for applications under elevated temperatures and cyclic loading, particularly in demanding industries like aerospace, biomedical, and turbine manufacturing^1,2^. These alloys, such as Hastelloy X, Inconel 718, and Inconel 625, exhibit outstanding yield strength and extended fatigue life, making them indispensable in these sectors, and their thermal stability allows them to maintain structural integrity even under extreme conditions^3,4^. While these alloys make up over 50% of the total weight of superalloys used in the industry, their excellent ductility at cryogenic temperatures, due to their face-centered cubic (FCC) crystal structure, also enhances their suitability for advanced applications such as superconducting materials, space rocket propulsion, and cryogenic storage^5,6^. Despite their strength and durability, nickel-based superalloys like Hastelloy X and Inconel are not typically used in long-term biomedical implants due to nickel-induced allergic reactions and biocompatibility concerns^7^. However, they are used in niche biomedical applications, particularly in surgical instruments and medical tooling, where exceptional strength, durability, and corrosion resistance are needed, but prolonged tissue contact is avoided^8^. Machining these alloys for such applications presents challenges, including rapid tool wear, surface degradation, high power consumption, and increased carbon emissions, due to their low thermal conductivity, high chemical reactivity, and elevated hardness^9^. These difficulties are further compounded in precision applications, where high cutting forces, material adhesion to tools, and notch wear result in significant machining challenges^10^. Additionally, the heat generated during cutting adversely affects both tool life and surface quality. To address these issues, manufacturers are exploring sustainable machining solutions that reduce energy consumption, cutting forces, and tool wear, leading to the development of various lubrication and cooling strategies, which are critically evaluated for cost-effectiveness and sustainability in medical tooling applications, as illustrated in Fig. 1.

Krolczyk et al.^12^ highlighted the importance of sustainable manufacturing strategies aimed at optimizing resource use while minimizing environmental impact. In a related study, Devillez et al.^13^ examined the dry machining of superalloys, focusing on tool wear by comparing the performance of carbide tools at various cutting conditions. The study revealed that adhesion, and the formation of built-up layers (BUL) on the workpiece surface, along with built-up edges (BUE) on the tool, were prevalent during the initial stages of machining. Under more aggressive machining conditions, increased adhesion led to the creation of BUE and BUL on the rake faces, ultimately resulting in tool notching. Similarly, Zhang et al.^14^ investigated the machining of nickel alloys under both minimum quantity lubrication (MQL) and dry cutting conditions, evaluating the workpiece surface condition and tool life. Their results showed that the tool life under MQL was extended by one and half times, besides the surface finish was significantly improved compared to dry medium. The improved tool wear was attributed to reduced friction, as the fine mist of the lubricant penetrated the tool-chip interface, resulting in lower chip adhesion, reduced cutting forces, and energy savings. Tamang et al.^15^ conducted a detailed study on the impact of MQL versus dry cutting techniques on cutting power and tool wear when machining Inconel 825. Their findings revealed that MQL significantly decreased cutting power by 8.47%, surface roughness by 10.41%, and tool wear by 16.57% in comparison to dry machining. In light of the increasing emphasis on sustainability, the adoption of biodegradable vegetable oils is suggested. Nevertheless, the limited flow rates and inadequate porosity of these vegetable oils have prompted researchers to investigate alternative cooling strategies, focusing on the reduction of coolant usage.

In the realm of machining nickel-based superalloys, the application of MQL utilizing nanofluid-based oils has proven effective in managing the thermal load induced by friction and shear forces within the machining zone. The incorporation of nanofluids significantly augments thermal conductivity and, due to their inherent tribological effects, presents a superior alternative for MQL systems^16^. Till now, a diverse array of nanoparticles are employed to mitigate friction by reducing the interaction between the tool-workpiece. The crystal structure, morphology, and dimensions of these nanofillers play a crucial role in determining their lubricating efficacy. Integrating these nano-additives into vegetable-based lubricants enhances their performance, thereby decreasing the reliance on mineral lubricants by decreasing friction and temperature, and improving tool longevity and surface finish^17^. Notably, Prasad^18^ demonstrated that the incorporation of graphene nano-additives in the machining of superalloy, in varying concentrations (0.1–0.5%), led to marked reductions in tool wear and operational temperature, with the most significant improvement observed at a 0.3% concentration, ultimately enhancing surface quality. Naresh Babu et al.^19^ conducted a comparative study evaluating the performance of MQL-based graphene nanofluids against conventional flood cooling and dry cutting conditions. Their findings indicated that MQL with graphene nanofluids outperformed both flood cooling and dry cutting techniques in terms of surface finish, tool life, and cutting temperature reduction. The enhanced performance was accredited to the excellent cooling and lubrication characteristics of MQL, making it a more efficient and sustainable choice compared to traditional flooded cooling. Danish et al.^20^ developed graphene-reinforced sunflower oil MQL machining of Inconel 718 to address heat generation in high-speed machining. The study compared the performance of this nano-green oil under dry, flooded, sunflower oil, and 0.7% graphene-reinforced sunflower oil conditions. The nano-oil reduced surface roughness by 49%, cutting force by 25%, cutting temperature by 31%, and tool wear by 20% compared to dry machining, also outperforming flooded lubrication. Elemental analysis identified adhesion as the primary wear mechanism in all conditions, highlighting the potential of graphene-enhanced vegetable oils for improving machining efficiency and sustainability. While the performance benefits of nanofluid-based lubricants are well established, the safety of nanoparticles for workers and the environment remains under study. Prolonged exposure to airborne nanoparticles may pose health risks due to their ability to penetrate biological membranes and accumulate in organs. Environmental concerns also exist over their persistence and bioaccumulation. Thus, proper handling, risk assessment, and regulatory compliance are essential to ensure safe use in machining applications.

Cryogenic cooling has emerged as a transformative and sustainable technique for enhancing the machinability of difficult-to-cut materials such as superalloys. This technique involves the application of cryogens—primarily liquid nitrogen (LN₂) and carbon dioxide (CO₂)—at subzero temperatures directly to the cutting zone, thereby significantly lowering the cutting temperature and mitigating thermal-induced tool wear. Few years ago, Grguras et al.^21^ studied robotic drilling of Ti6Al4V in aerospace, focusing on dry drilling’s tool wear and heat issues. They used LCO_2_ + MQL for cooling/lubrication, varying flow rates to observe effects on thrust force, torque, and chip morphology. Results showed LCO_2_ flow rate impacts thrust force, while MQL affects torque. LCO_2_ + MQL improved chip breakability and reduced tool adhesion compared to dry drilling. Shokrani et al.^22^ compared dry, flood, and cryogenic environments during milling of Ti-6Al-4 V alloy. Their findings revealed that cryogenic and flood cooling led to 30–40% lower surface roughness than dry cutting, while cryogenic cooling specifically minimized plastic deformation and chip re-deposition due to its localized and rapid cooling effect. However, despite these advantages, cryogenic machining often suffers from inadequate lubrication, which can limit its tribological performance. To overcome this limitation, hybrid cooling strategies have been explored. Shokrani et al.^23^ proposed a novel cryogenic-assisted MQL technique, which synergistically combines the cooling effectiveness of LN₂ with the lubrication benefits of MQL. Their study reported up to a 30-fold increase in tool life during the machining of Ti-6Al-4 V compared to conventional flood cooling. Arafat et al.^24^ investigate alternatives to mineral oil-based cutting fluids, particularly MQL with supercritical CO_2_ for surface grinding. Their study reveals that MQL-scCO_2_ results in similar or lower forces and cutting power compared to conventional emulsion cooling but leads to increased surface roughness. The paper highlights the need for further research on nozzle design, thermal analysis, and wheel dressing, with preliminary energy performance indicators suggesting a potential for cleaner production. Khanna et al.^25^ investigated sustainable drilling of Ti6Al4V ELI implants using electrostatic MQL (EMQL) and cryogenic LCO₂ coolants. Due to titanium’s poor machinability, proper lubrication is essential. SEM and EDS analyses revealed that LCO₂ offered superior hole quality, 42–71% lower surface roughness (Rz), and 416% longer tool life than EMQL. Defects like adhesion, smearing, and microfractures appeared under both methods, but EMQL showed more severe wear and chip damage. Overall, LCO₂ proved more effective and eco-friendly for machining Ti6Al4V. Agrawal et al.^26^ investigated the machinability of Ti–6Al–4 V under wet and cryogenic conditions at cutting speeds of 70–110 m/min. Cryogenic turning showed lower crater wear, power consumption (up to 23.4%), surface roughness (up to 22.1%), machining cost (up to 27%), and carbon emissions (up to 22%) compared to wet turning, especially at higher speeds, with tool life improved by up to 125%. The study highlights cryogenic machining as a more sustainable alternative to conventional methods. Khanna et al.^27^ investigated cryogenic machining as a sustainable approach for drilling Inconel 718, analyzing thrust force, torque, tool wear, chip morphology, and hole quality. Results showed that cryogenic drilling significantly reduced temperature-induced tool wear, lowering torque by up to 30%, and improving tool life by 87.5%. It also enhanced hole quality with reductions in circularity (51%), cylindricity (77%), and surface roughness (48%) compared to dry drilling, demonstrating its effectiveness in improving machinability and sustainability. Pereira et al.^28^ highlighted the machining difficulties of Inconel 718, a heat-resistant alloy with low machinability, high strength at elevated temperatures, and strain hardening behavior, leading to high cutting forces and temperatures. To address both technical and environmental challenges, they proposed a Cryo-MQL approach using internal CO₂ cooling and external MQL during milling. Compared to conventional emulsion and standalone MQL, this method improved tool life by 57% and 120%, respectively, demonstrating enhanced performance and sustainability. Recently, Khanna et al.^29^ highlight the challenges of machining Inconel 625 due to its high strength, hardness, and heat resistance, leading to rapid tool wear, poor surface finish, and high energy consumption. This study focuses on drilling Inconel 625, a common nickel-based superalloy, using two travel speeds (175 mm/min and 185 mm/min) and sustainable lubrication/cooling strategies—EMQL and cryogenic LCO_2_. Results show that the sample fabricated at 185 mm/min exhibits lower tool wear, surface roughness, thrust force, and power consumption. LCO_2_ reduces tool wear by 35%, surface roughness by 27%, thrust force by 15%, and power consumption by 4% compared to EMQL.

The existing body of literature highlights that the dry, MQL, and cryogenic cooling techniques represent the predominant strategies investigated within sustainable machining research. Nonetheless, a conspicuous void remains in the discourse surrounding the integrated assessment of technological, economic, and environmental dimensions, particularly in the context of hard-to-machine materials like superalloys. In this regard, the present study contributes a novel perspective by not only optimizing the cutting parameters but also systematically evaluating the influence of various lubrication and cooling environments on the sustainable machining of Hastelloy X. Distinct from previous studies that have primarily focused on machining performance indicators alone, this study uniquely emphasizes the economic implications and energy efficiency aspects associated with sustainable machining practices. An established costing model was employed to ascertain the per-unit production costs, while an energy consumption model was applied to approximate the total energy expenditure of the machine tool. The insights garnered from this investigation are of considerable significance, as they delineate the optimal lubrication and cooling conditions conducive to sustainable machining, thereby enhancing productivity while concurrently mitigating energy consumption and machining costs. Thus, this study offers a holistic and comprehensive framework for sustainable machining of superalloys, bridging an important gap in the existing literature and contributing valuable guidelines for industry practitioners aiming for technologically efficient, economically viable, and environmentally responsible manufacturing.

## Materials and methods

### Experimental details

This study used 60 × 180 mm Hastelloy X bars made from a commercially available nickel-based superalloy. The primary elemental composition of the alloy, as provided in the material test certificate supplied by the vendor, is presented in Table 1. The turning experiments were conducted using carbide inserts (ISO designation: CNGG 120404 ML), which feature a fine-grain tungsten carbide (WC) substrate with a multi-layer TiAlN-TiN coating (coating thickness of 2–5 microns) for enhanced wear resistance and heat tolerance. Machining trials were carried out on a CNC-enabled lathe (MTAB MAXTURN PLUS) having a spindle power of 3.7 kW. The detailed specifications of the machine and tooling are provided in Table 2. To investigate the effect of different cooling and lubrication environments on machining performance, experiments were conducted under four distinct cutting environments: Dry, MQL, LN₂ cooling, and CO₂-snow cooling.

In the dry cutting environment, no external coolant or lubricant was applied during machining. This condition served as a baseline reference to evaluate the comparative performance of sustainable cooling and lubrication techniques. The experiments were performed under identical cutting parameters used in other environments to ensure consistency.

For the MQL setup, sunflower oil was selected as the base fluid owing to its superior oxidative stability, excellent lubricating properties, lower viscosity, and enhanced biodegradability compared to other vegetable-based oils, as reported in previous literature^20^. The thermo-physical properties of sunflower oil are presented in Table 3. The oil was supplied through a single-nozzle system (nozzle diameter: 2 mm), operated at a 6 bar air pressure and a 2.5 ml/min of flow rate. The nozzles were strategically positioned 25 mm from the machining zone to effectively target the tool-workpiece interface.

In the case of cryogenic machining, a specially designed LN₂ delivery system was employed to ensure the effective supply of liquid nitrogen directly at the cutting zone. LN₂, maintained at −196 °C, was delivered through a 2.5 mm diameter nozzle under 6 bar pressure and a 2 kg/min of flow rate. The nozzle was positioned very close to the cutting zone (at a distance of 25 mm) to minimize the evaporation loss of LN₂ before reaching the cutting zone. Additionally, the controlled flow rate and insulated delivery line ensured that LN₂ was delivered in its liquid state at the cutting interface. The presence of liquid-phase nitrogen at the cutting zone was further verified through visual observation of frost formation, confirming the effectiveness of the cryogenic delivery system.

Furthermore, a CO₂-snow cooling system was integrated to improve localized cooling. The CO₂-snow was generated through the Joule-Thomson effect by the rapid expansion of liquid CO₂ stored at 55 bar pressure, using a nozzle of 0.5 mm diameter placed 25 mm from the cutting zone. The system operated at a flow rate of 0.2 kg/min, achieving a temperature of −78 °C at the cutting zone, as verified using temperature sensors and pressure gauges.

**Table 1:** Elements of Hastelloy X.

Element	Range
Nickel (Ni)	47.0–51.0%
Chromium (Cr)	20.0–22.0%
Molybdenum (Mo)	8.0–10.0%
Iron (Fe)	1.0–2.5%
Cobalt (Co)	1.0%
Tungsten (W)	1.0%
Carbon (C)	0.05%
Manganese (Mn)	1.0%
Silicon (Si)	0.5%
Phosphorus (P)	0.04%
Sulfur (S)	0.03%

**Table 2:** Cutting environment descriptions and parameters.

Category	Specifications
Machine	CNC lathe
Workpiece material	Hastelloy X (60 × 180 mm)
Lubri-cooling medium	Dry, MQL, Cryo-LN_2_, Cryo-CO_2_
Cutting insert	CNGG 120,404 ML (Grade: TT5080)
Cutting speed	80 m/min, 150 m/min
Feed	0.2 mm/rev, 0.4 mm/rev
Depth of cut	0.50 mm

**Table 3:** Thermo-physical characteristics of sunflower oil.

Property	Value
Density	0.920–0.925 g/cm³
Viscosity (at 40 °C)	30–35 cP
Specific Heat Capacity	1.96–2.0 J/g°C
Thermal Conductivity	0.16–0.18 W/m·K
Flash Point	232–250 °C
Pour Point	−10 to −6 °C
Boiling Point	232–250 °C
Smoke Point	227–235 °C
Refractive Index (at 20 °C)	1.47–1.48

### Response measurement

The average surface roughness (*R*_*a*_) of the machined area was meticulously assessed using a portable Perthometer (MahrSurf, USA). Measurements were conducted at intervals of 25, 50, 75, and 100 mm along the machined surface, with the roughness values averaged from four distinct observations. A Gaussian profile filter with a cut-off length of 0.8 mm was applied, in accordance with ISO 11,562 standards. This approach provided a reliable evaluation of the surface finish, capturing subtle variations in roughness across the machined surface. Besides, the tool wear was a critical parameter in evaluating the machining performance. The flank wear on the cutting inserts was thoroughly examined using a SEM (ARTCAM 130-MT-WOM, Japan). High-resolution images of the inserts were captured at various intervals of the machining process to monitor the progression of wear. The maximum flank wear was then determined by analyzing these images, allowing for a detailed assessment of wear mechanisms and the durability of the cutting tools under the given machining conditions. Note that each experiment was repeated three times under identical conditions to ensure repeatability and reliability of the results.

The total machining cost (*C*_*T*_) comprises electricity consumption cost (*C*_*E*_), cutting insert cost (*C*_*I*_), and lubricant cost (*C*_*L*_), as shown in Eq. (1). This study focuses on direct machining costs like electricity, tools, and lubricants, excluding overhead and disposal costs for simplicity, though a comprehensive analysis should include all relevant expenses for accuracy.

1 $$\:{C}_{T}={C}_{E}+{C}_{I}+{C}_{L}$$

*C*_*E*_ can be described by:

2 $$\:{C}_{E}=\:\frac{{C}_{UE}}{{\upeta\:}\times\:60\times\:1000}\times\:{T}_{M}\times\:({C}_{P}+{S}_{P})$$

Here, *C*_*UE*_ stands for the average energy cost per kilowatt-hour (₹6.19/kWh, based on the average electricity cost in India). *η* represents the machine’s efficiency, which is 85%. *C*_*P*_ refers to the cutting power, measured at 2216 W when operating at a speed of 100 m/min. *S*_*P*_ indicates the standby power consumption, which is approximately 1987 W.

The machining time (*T*_*M*_) is calculated using:

3 $$\:{T}_{M}=\:\frac{Length\:of\:cut+Machining\:allowance}{Feed\:rate\times\:RPM}$$

The length of the cut is determined by:

4 $$\:Length\:of\:cut=\frac{Market\:price\:of\:cutting\:insert}{Tool\:life}\times\:{T}_{M}$$

*C*_*I*_ plays a key role in machining, affecting both cost and productivity. Inserts are replaced when wear reaches 0.3 mm, as performance drops sharply beyond this point. The initial cost of each cutting insert is considered as ₹850.

Furthermore, the lubricant cost, *C*_*L*_ is given by:

5 $$\:{C}_{L}=Lubricant\:or\:coolant\:cost\times\:Flow\:rate\:of\:lubricant\:or\:coolant\times\:{T}_{M}$$

The price of sunflower oil is estimated at ₹250 per liter, while cryogenic LN_2_ and cryogenic CO_2_ are priced at ₹320 and ₹350 per liter, respectively.

Furthermore, energy consumption analysis is crucial due to three key factors: (a) energy costs account for nearly half of production expenses, (b) the global push for alternative energy sources, and (c) the environmental harm caused by carbon emissions. This study uses Eq. (6) to explore ways to create a more energy-efficient cutting environment.

6 $$\:{E}_{C}=\:\frac{{F}_{R}\times\:{V}_{C}\times\:T}{1000}$$

**Fig. 2:**
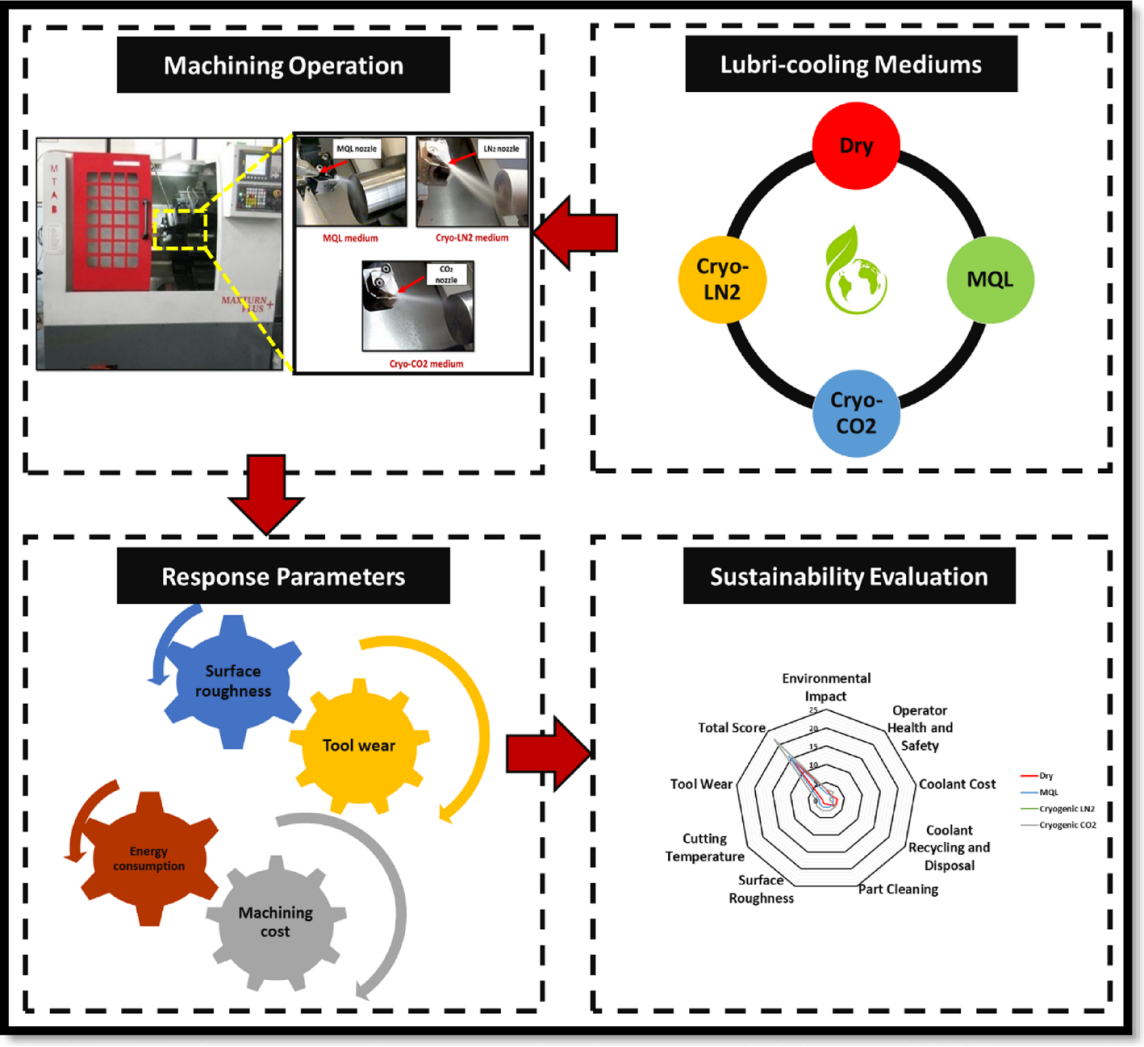
Comprehensive illustration of the methodology employed in this study.

Here, *F*_*R*_ is the resultant cutting force (Eq. (7)), *V*_*C*_ is cutting speed, *T* is cutting time, and *E*_*C*_ is energy consumption. A detailed overview of the methodology used in this study is shown in Fig. 2.

7 $$\:{F}_{R}=\:\sqrt{{{F}_{x}}^{2}+{{F}_{y}}^{2}+{{F}_{z}}^{2}}$$

Where, *F*_*x*_, *F*_*y*_, and *F*_*z*_ are forces in the *x*, *y*, and *z* directions, respectively.

It is important to mention that the cutting forces during the turning process were measured using a Kistler Type 9121 piezoelectric dynamometer, which recorded forces in the *x*, *y*, and *z* directions. The dynamometer was mounted on the tool post, and the force signals were captured throughout the machining operation. These values were then used in the *F*_*R*_ calculation.

## Results and discussion

### Impact of lubrication/cooling mediums on tool wear

**Fig. 3:**
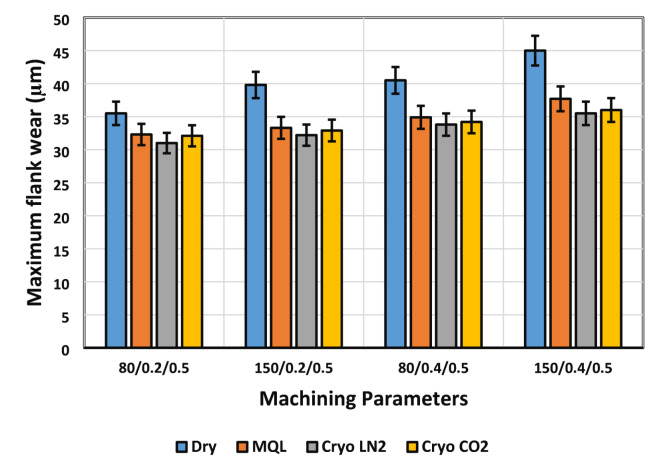
Influence of machining inputs on tool degradation under varied lubri-cooling environments.

Tool wear is a critical factor in cutting operations, as it directly influences energy consumption, carbon emissions, and overall operational costs, particularly under high wear conditions. Excessive tool wear not only increases expenses but also undermines ecological sustainability, emphasizing the need for effective wear mitigation strategies. Figure 3 illustrates the influence of various machining parameters on tool wear values across different lubri-cooling environments. For an instance, tool wear was measured after 15 min of machining under each lubricating condition, with cutting parameters — a speed of 150 m/min, feed rate of 0.4 mm/rev, and depth of cut of 0.50 mm — held constant throughout. The results clearly demonstrate that Cryo-LN_2_, Cryo-CO_2_, and MQL conditions significantly outperform dry machining in reducing tool wear. Specifically, flank wear reductions of 21.11%, 20%, and 16.22% were achieved under Cryo-LN_2_, Cryo-CO_2_, and MQL, respectively, relative to dry conditions, with Cryo-LN_2_ yielding the least tool wear. Although the lowest flank wear was observed at a cutting speed of 80 m/min and a feed rate of 0.2 mm/rev, this optimal performance supports earlier findings that higher cutting speeds produce more heat and subsequently elevate wear rates. Furthermore, increased feed rates can intensify tool vibrations, making high-speed operations less ideal when minimizing tool wear is crucial^30^.

**Fig. 4:**
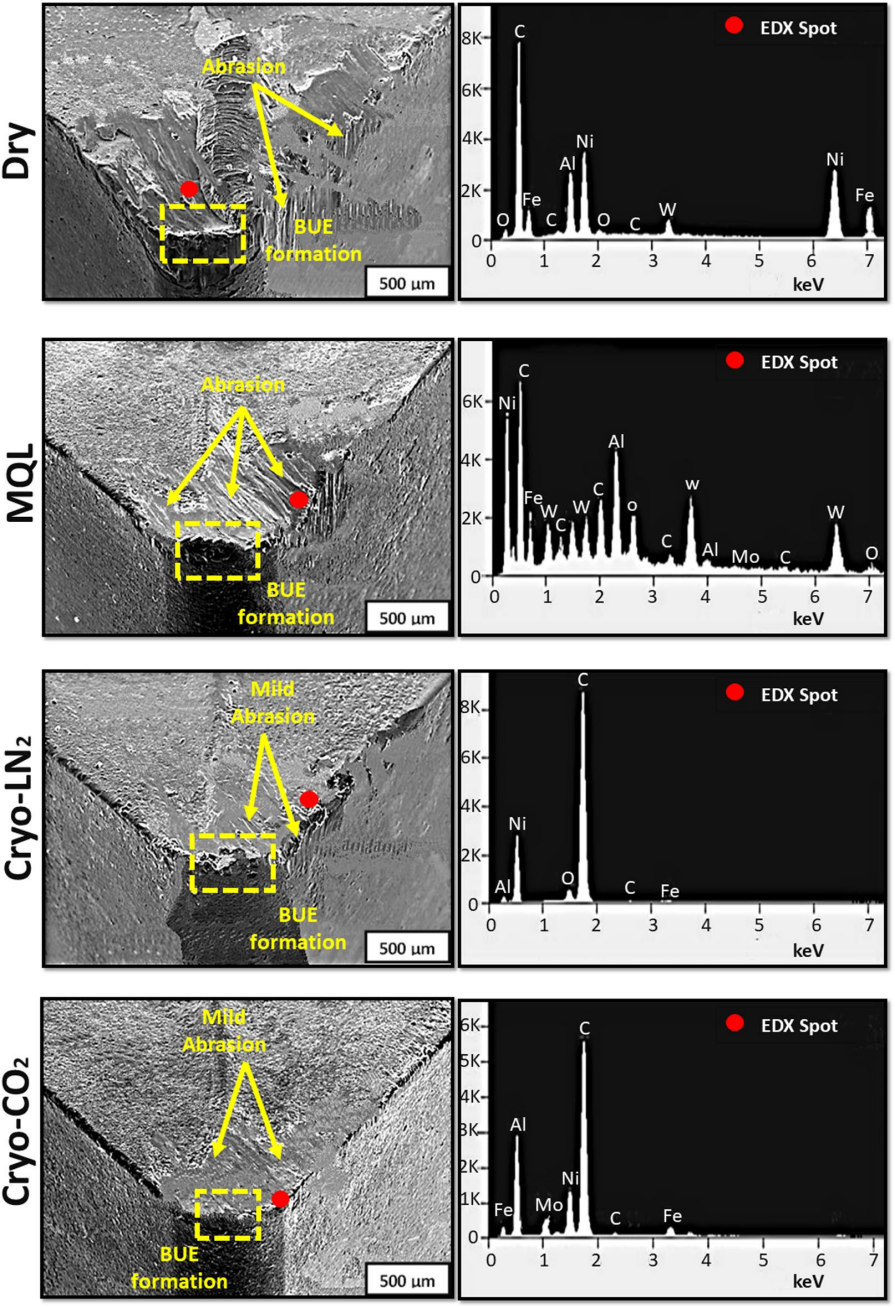
SEM analysis of cutting edges in different lubri-cooling environments.

SEM and EDX images (Fig. 4) reveal that adhesion constitutes the predominant wear mechanism across all lubri-cooling conditions investigated. The research indicates that heightened adhesion significantly increases the probability of BUE formation at the cutting edges. Adhesive wear manifests as the coating material is eroded from the insert, thereby exposing the underlying substrate. This phenomenon was uniformly observed in all experimental conditions, with adhesion being primarily influenced by elevated pressure on the rake face. Increased cutting speeds and high temperature at the tool-chip interface further exacerbate this condition, softening the workpiece material and intensifying its tendency to adhere to the tool surface. Such conditions promote the diffusion of material from the workpiece onto the tool, forming a strongly bonded layer.

Additionally, abrasion was observed on the tool surfaces across all cooling environments, with large particles in the workpiece material creating grooves on the flank faces. The interaction between the tool and the chip, which leads to abrasion, further compromises the tool’s durability. Abrasion not only creates visible grooves but also accelerates the removal of the tool’s coating layer, thereby exposing the substrate to further wear mechanisms. The abrasive particles embedded in the workpiece material may include hard inclusions such as carbides or oxides, which exert significant mechanical stress on the tool’s surface. As these particles pass over the tool’s surface under elevated temperatures and cutting forces, they plow through the coating and substrate material, leading to rapid wear progression. In severe cases, this abrasive action can result in micro-cracks and localized material removal, weakening the tool structure. The combined effects of adhesion and abrasion create a synergistic wear mechanism that significantly shortens the tool’s lifespan, particularly under extreme cutting conditions.

### Impact of lubrication/cooling mediums on average surface roughness

**Fig. 5:**
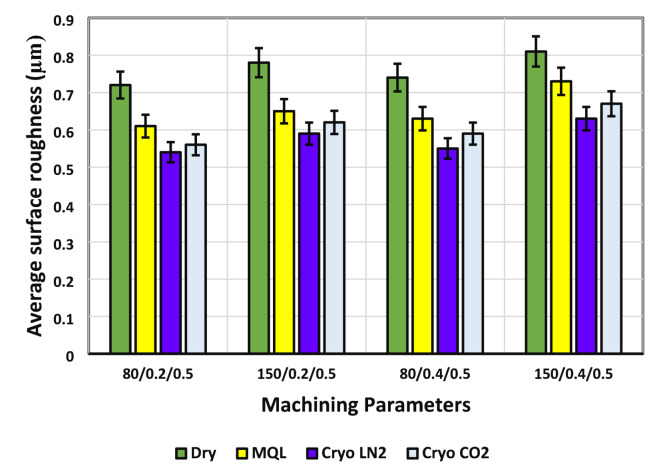
Variation in Average Surface Roughness with Different Lubri-Cooling Mediums.

The choice of various lubri-cooling mediums significantly influences surface roughness during machining processes. Achieving a smooth and flawless finish is highly dependent on selecting the optimal machining parameters, which in turn ensures precise dimensional accuracy of the workpiece. Previous research indicates that factors such as nose radius and cutting feed play a crucial role in relation to the cutting tool, ultimately affecting surface roughness. For superior machining performance, it is essential to produce discontinuous chips while avoiding the formation of BUE during the machining of superalloys^31^. In the present study, surface roughness and surface morphology measurements were performed under identical cutting conditions (cutting speed of 150 m/min, feed rate of 0.4 mm/rev, and depth of cut of 0.50 mm) after 15 min of machining, corresponding to a specific stage of tool wear. This approach ensures consistency in evaluating the effect of different lubri-cooling mediums on surface quality. Figure 5 illustrates how average surface roughness varies with cutting parameters and different lubri-cooling mediums. As cutting speed increases, surface roughness decreases, although a higher feed rate tends to elevate roughness. Interestingly, surface roughness is more sensitive to changes in feed rate than to cutting speed. Different lubricating and cooling conditions lead to considerable variations in surface roughness, with significant reductions observed under Cryo-CO_2_, Cryo-LN_2_, and MQL regimes. A slight difference in surface roughness was noted when comparing Cryo-LN_2_ and Cryo-CO_2_ at the highest machining parameter settings. Specifically, surface roughness reductions under Cryo-CO_2_, Cryo-LN_2_, and MQL conditions were 22.22%, 25%, and 15.28%, respectively, compared to dry machining at a cutting speed of 80 m/min and a feed rate of 0.2 mm/rev. Similarly, at a higher cutting speed of 150 m/min and a feed rate of 0.4 mm/rev, the reductions in surface roughness were 17.28%, 22.22%, and 9.88%, respectively.

**Fig. 6:**
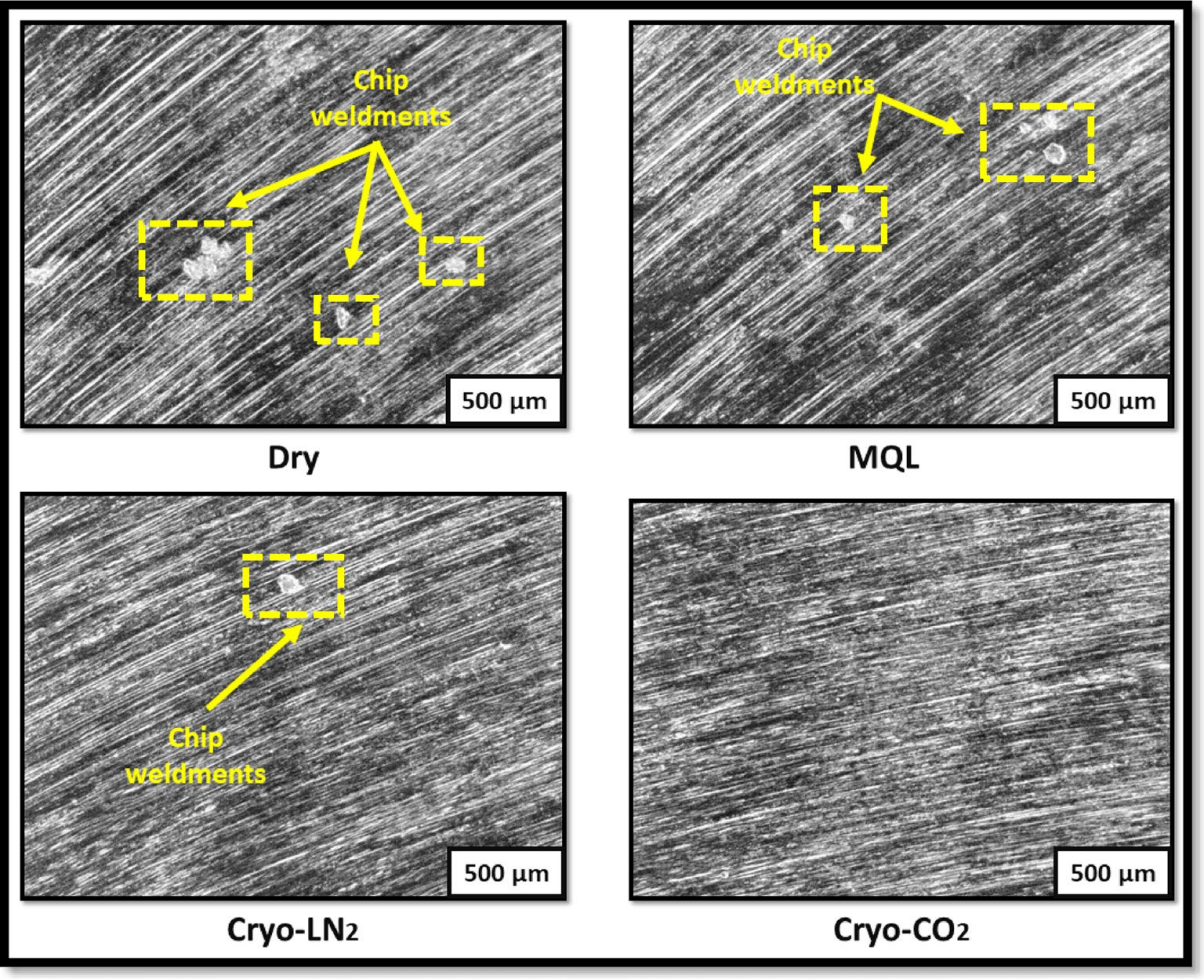
Micrographic images of cutting edges in different lubri-cooling environments.

The comparative micrographic images of the machined surface of Hastelloy X under different lubri-cooling environments are presented in Fig. 6. These observations correspond to the same cutting conditions employed for tool wear analysis. The images clearly reveal the presence of chip weldments on the machined surfaces in both dry and MQL environments. Conversely, fewer chip weldments are observed under the Cryo-LN_2_ environment, and notably, no chip weldments are visible in the Cryo-CO_2_ environment. The occurrence of chip weldments in dry and MQL conditions can be primarily attributed to the elevated temperatures generated during the cutting process. In dry machining, the absence of any cooling or lubricating medium causes the heat generated at the tool-workpiece interface to remain localized, leading to thermal softening or even partial melting of the chips. Consequently, these softened chips adhere to the tool or workpiece surface, resulting in the formation of chip weldments. Although MQL provides a minimal amount of lubricant, it is insufficient to completely dissipate the heat generated, leading to some degree of chip adhesion and weldment formation, albeit less than that observed in dry machining. In contrast, cryogenic cooling significantly alters this behavior. The application of cryogenic fluids, particularly LN_2_, substantially reduces the temperature at the cutting zone, thereby minimizing heat generation during machining. This effective cooling prevents thermal softening of the workpiece material and mitigates the formation of chip weldments. Remarkably, the Cryo-CO_2_ environment exhibited the most effective suppression of chip weldments, highlighting its superior cooling and lubrication capabilities.

**Fig. 7:**
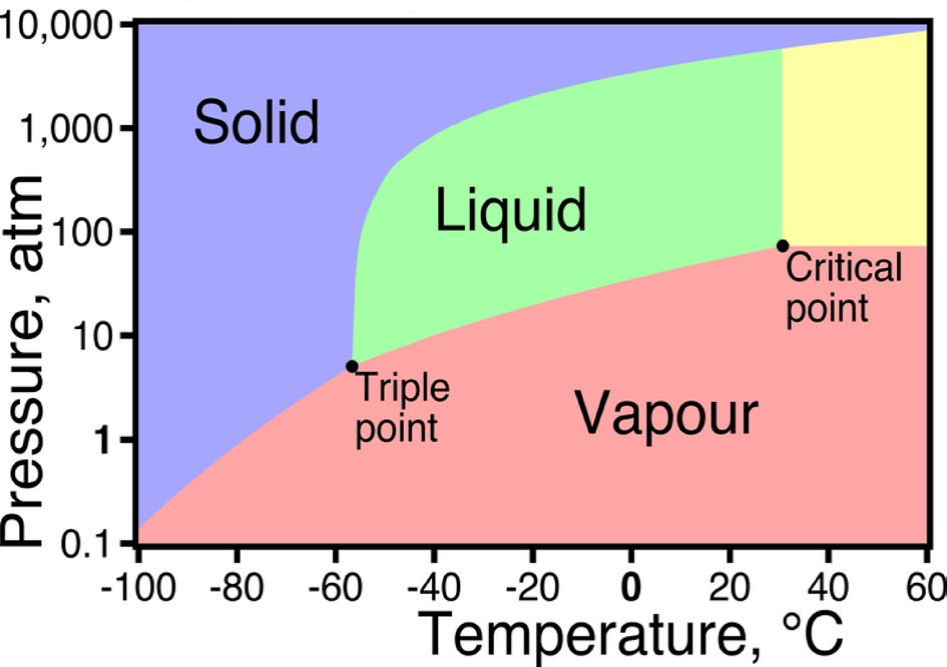
Phase diagram of CO_2_.

The most striking observation, however, is the absence of chip weldments in the Cryo-CO₂ environment. This phenomenon can be attributed to the unique thermodynamic behaviour of CO₂ during its phase transition, as illustrated in Fig. 7. As evident from the CO₂ phase diagram, at atmospheric pressure (~ 1 atm), CO₂ does not undergo a liquid-to-gas transition; instead, it sublimates — directly transforming from solid to vapour — bypassing the liquid phase entirely. This sublimation process demands a substantial amount of latent heat absorption from the surrounding environment. When CO₂ is applied as a cooling medium in machining, either in its solid (dry ice) or pressurized liquid form, it rapidly expands upon exposure to atmospheric conditions, undergoing sublimation (in the case of solid CO₂) or flash evaporation (in the case of pressurized liquid CO₂). Both these processes result in efficient heat extraction from the cutting zone due to the high latent heat of sublimation or vaporization of CO₂. Consequently, this leads to a significant reduction in the cutting temperature, thereby minimizing the chances of BUE formation and chip weldments on the cutting tool surface. Thus, the excellent cooling capacity of CO₂, governed by its phase transformation behaviour, plays a pivotal role in enhancing the machining performance.

### Impact of lubrication/cooling mediums on energy consumption

**Fig. 8:**
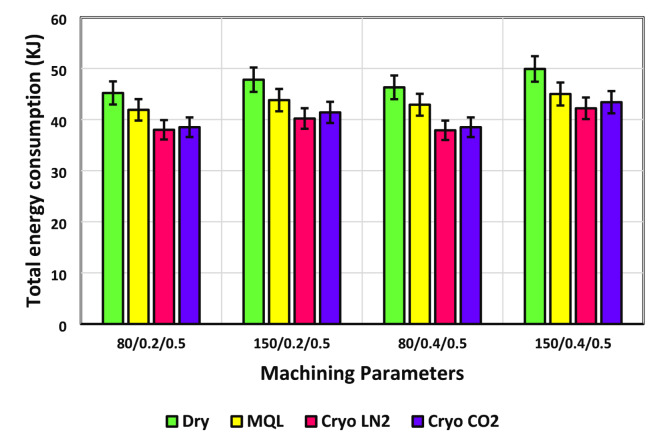
Energy consumption in different lubrication/cooling conditions.

Given the finite nature of fossil fuel reserves and the inevitable decline of non-renewable energy sources, it is imperative to prioritize energy efficiency alongside product quality in machining processes to ensure sustainable production. Machine tools, which are responsible for a significant share of energy consumption in the industrial sector, are central to this objective. A considerable amount of the total energy consumed in manufacturing is attributed to the machining process itself. This energy demand escalates when machining materials that are difficult to process or possess low machinability. The data on energy consumption under sustainable lubrication and cooling regimes, as shown in Fig. 8, provides valuable insights for reducing overall energy use. A notable trend is the increase in energy consumption with higher cutting speeds, largely due to the variation in energy usage with spindle speed. As spindle acceleration intensifies, the motor requires more power, leading to greater energy draw. Additionally, non-machining activities, especially tool-changing operations, demand more energy at elevated cutting speeds due to accelerated tool wear, necessitating more frequent tool replacements, thereby increasing electrical energy consumption.

**Fig. 9:**
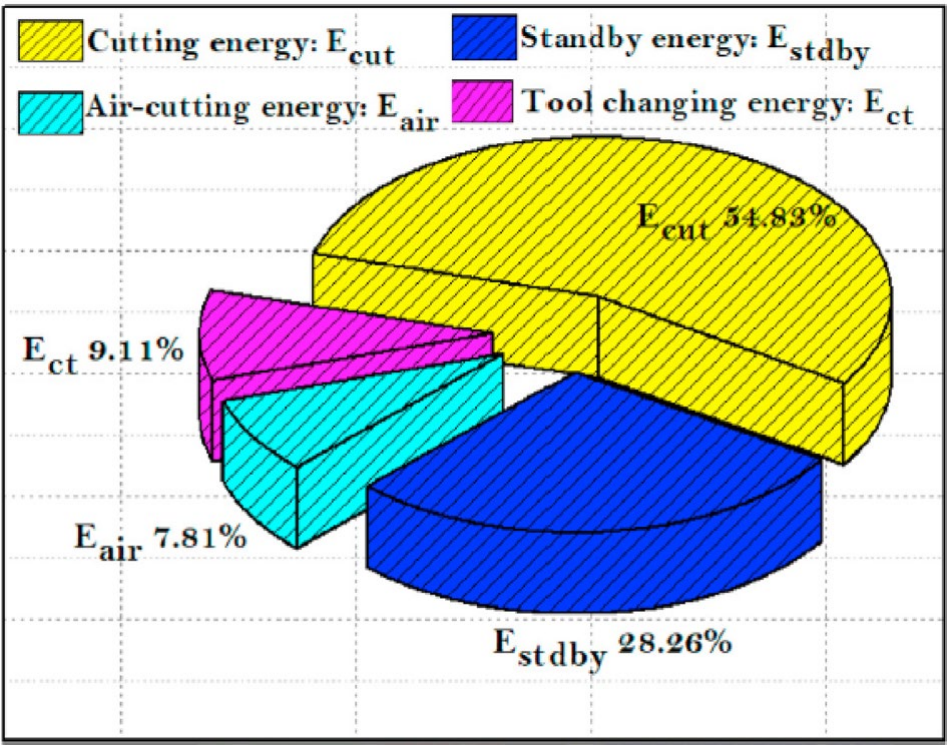
Energy distribution across various phases of the machining operation^32^.

While cutting speed is a key determinant of energy use, an increase in feed rate also contributes to a gradual rise in total energy consumption. During the machining process, total energy consumption is closely linked to the material removal rate (MRR), which depends on the feed rate, cutting speed, and depth of cut. Higher feed rates tend to increase surface roughness, indicating a greater distance between peaks on the machined surface. This necessitates more tool movement per revolution, thereby consuming more energy. Consequently, the interplay of feed rate and cutting speed has a significant impact on costs, time, effort, and energy consumption. Figure 8 also shows the relationship between total energy consumption and various lubrication and cooling strategies across different speed-feed combinations. The findings indicate that energy consumption is highest during dry cutting, followed by decreasing levels with Cryo-LN_2_, Cryo-CO_2_, and MQL techniques. Although dry cutting eliminates coolant use, reducing health and environmental risks, it results in high energy consumption, which challenges sustainable machining efforts. Compared to dry turning, the implementation of MQL, Cryo-LN_2_, and Cryo-CO_2_ resulted in energy savings of 7.30%, 15.93%, and 14.82%, respectively, at the lowest speed and feed rate. A similar pattern was observed at the highest speed-feed combination, with energy consumption reductions of 9.82%, 15.43%, and 13.03% for MQL, Cryo-CO_2_, and Cryo-LN_2_, respectively, when compared to dry turning. This demonstrates that cryogenic cooling using LN_2_ is more effective in lowering energy consumption than traditional cooling methods. In light of these findings, both cryogenic and MQL cooling methods are recognized as environmentally friendly and energy-efficient techniques for machining. However, a critical aspect of energy management in machining is the assessment of energy consumption at each stage of the process. This allows for the identification of key energy-intensive phases, enabling targeted energy-saving strategies. Gupta et al.^32^ illustrated the distribution of energy consumption during different phases of the machining process using a pie chart (Fig. 9). They found that a significant portion of total energy is consumed during tool changes, idle times, and air cutting. Therefore, minimizing the duration of these phases is essential for achieving meaningful energy reductions when they represent a substantial share of overall energy consumption. In summary, specific cutting parameters—such as a cutting speed of 80 m/min and a feed rate of 0.2 mm/rev, combined with Cryo-LN_2_ cooling—are recommended to minimize energy consumption during cutting operations.

### Impact of lubrication/cooling mediums on machining cost

**Fig. 10:**
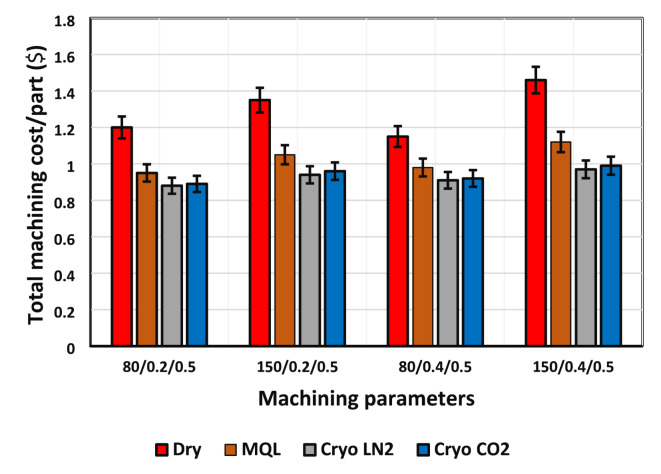
Total Machining Cost per Part for Various Parameter Combinations.

Currently, a primary goal for manufacturing facilities is to accelerate production while minimizing costs and ensuring high-quality output. Achieving the desired production levels during cutting operations often necessitates increasing both cutting speed and feed rate. However, this escalation in operational parameters leads to higher heat generation and accelerated tool wear, thereby shortening tool life. Consequently, frequent tool replacements are required, which not only reduces overall productivity due to increased idle time but also raises production costs. This frequent replacement of cutting tools further contributes to elevated. The comprehensive cost of machining is intricately affected by variables such as actual machining time, periods of inactivity, the total duration of machining operations, and intervals for tool replacements. To make cutting tools last longer, it’s important to fine-tune the machining settings and use eco-friendly methods for cooling and lubrication. This research focuses on comparing the cost of making parts using different machining setups, especially those that involve sustainable cooling and lubrication. A bar graph is used to clearly show how the production costs change under these various conditions. Figure 10 illustrates the cost per part, revealing that a cutting speed of 80 m/min combined with a feed rate of 0.2 mm/rev is notably advantageous for reducing costs. Notably, the use of Cryo-LN_2_ results in components that are 1.12%, 7.37%, and 26.67% less expensive than those produced with Cryo-CO_2_, MQL, and dry machining methods, respectively, when applied at a low-speed/feed combination. The Cryo-LN_2_’s ability to extend tool life, compared to dry, Cryo-CO_2_, and MQL methods under similar cutting conditions, is noteworthy. LN_2_ enhances cooling efficiency by preventing tool wear and keeping the machined surfaces clean and dry. This approach eliminates additional tasks, such as cleaning the finished products, thus contributing to overall cost savings.

### Pugh matrix-based sustainability assessment

The machining of high-performance materials like Hastelloy X presents unique challenges due to their toughness and high-temperature resistance. Traditional cooling and lubrication methods, such as flood cooling, often lead to environmental and health concerns due to the use of cutting fluids. In recent years, alternative methods such as dry machining, MQL, Cryo-LN_2_, and Cryo-CO_2_ have gained attention for their potential sustainability benefits. This evaluation employs the Pugh matrix to compare these methods based on various sustainability criteria. The Pugh matrix, also known as the decision matrix, is a tool used to evaluate and compare different options against a set of criteria^33,34^. Each option is scored based on how well it meets each criterion, allowing for a systematic comparison as shown in Table 4.

**Table 4:** Pugh matrix-based comparison.

Criteria	Weight	Dry	MQL	Cryo-LN_2_	Cryo-CO_2_
Environmental Impact	2	3	2	3	3
Operator Health and Safety	2	2	2	3	3
Coolant Cost	1	3	2	1	1
Coolant Recycling and Disposal	1	3	2	3	3
Part Cleaning	1	1	2	3	3
Surface Roughness	1	1	2	3	3
Cutting Temperature	1	1	2	3	3
Tool Wear	1	1	2	3	3
**Total Score**		15	16	22	22

The following provides an overview of the criteria used in the Pugh matrix:

- Environmental impact: Dry and cryogenic mediums score the highest due to their minimal environmental footprint. MQL also performs well, as it reduces the volume of coolant used.
- perator health and safety: Cryogenic methods again excel, as they do not involve harmful chemicals. MQL and dry machining present fewer health risks and require careful handling.
- Coolant cost: Dry machining is the most cost-effective option, as it eliminates coolant costs entirely. MQL has moderate costs, while cryogenic methods can be expensive due to the need for specialized equipment and handling.
- Coolant recycling and disposal: Dry and cryogenic methods score high as they do not produce waste fluids that require disposal.
- Part cleaning: Cryogenic cooling methods provide superior part cleanliness due to their effective cooling capabilities, followed by MQL. Dry machining may leave residues that require additional cleaning.
- Surface roughness: Cryogenic methods yield the best surface finish, essential for high-performance applications. MQL also provides good results, while dry machining may lead to poorer surface quality.
- Cutting temperature: Cryogenic cooling methods excel in maintaining lower cutting temperatures, which is crucial for machining tough materials like Hastelloy X. MQL also performs well, while dry machining can lead to higher temperatures.
- Tool wear: Cryogenic methods significantly reduce tool wear, extending tool life and reducing costs. MQL also shows reduced wear compared to dry machining, which can lead to increased tool degradation.

**Fig. 11:**
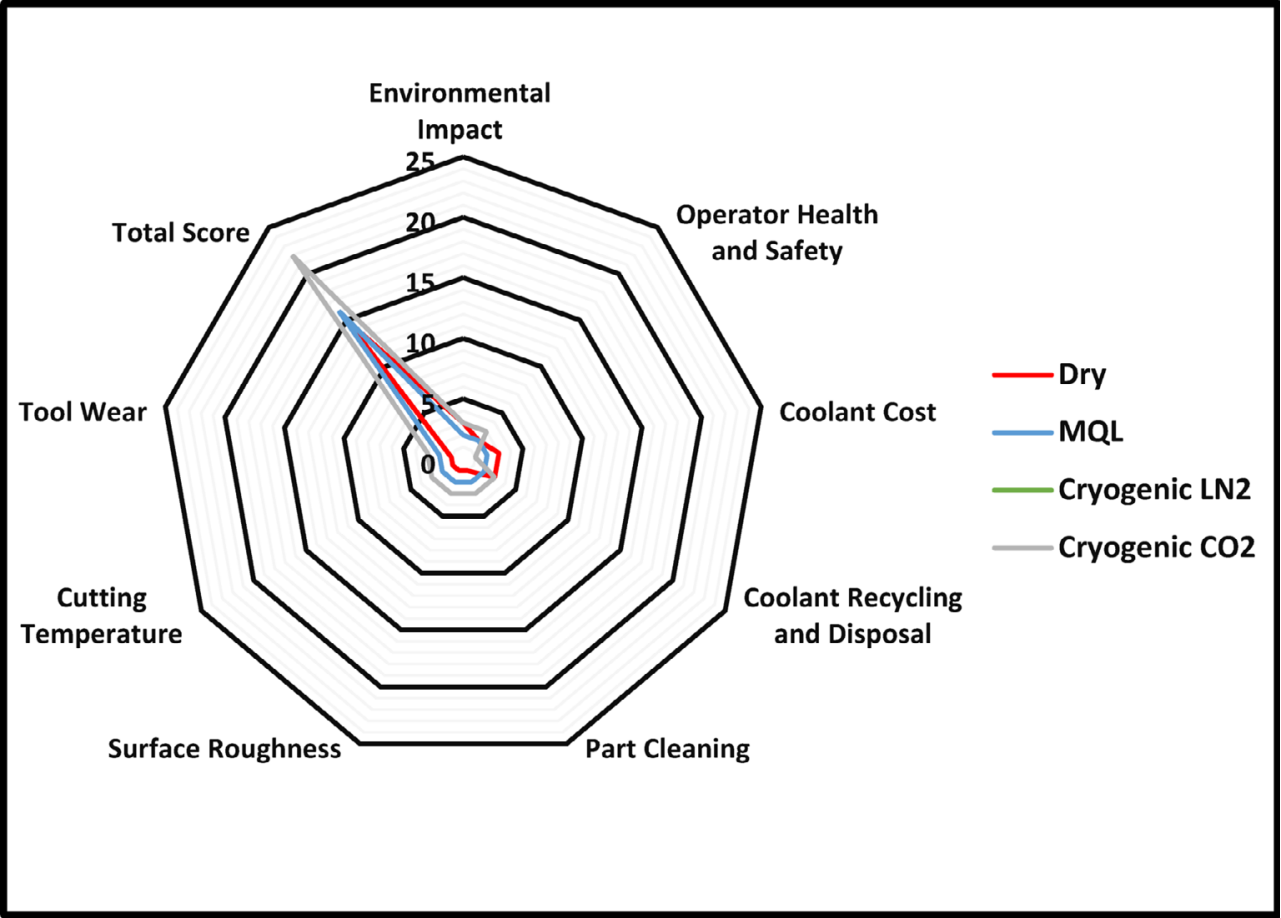
A radar chart of sustainability evaluation.

The Pugh matrix evaluation reveals that cryogenic cooling methods are the most sustainable options for machining Hastelloy X, offering significant advantages in environmental impact, operator safety, and machining performance. While dry machining is cost-effective, it may compromise surface quality and tool life (Fig. 11). The findings suggest that adopting cryogenic cooling can lead to cleaner production processes, improved machinability, and reduced environmental impact, aligning with the principles of sustainable manufacturing. Future research should focus on optimizing these methods and exploring their application in other high-performance materials.

## Conclusions

The present study comprehensively investigated the sustainable machining of Hastelloy X — a nickel-based superalloy widely employed in aerospace, chemical, and biomedical industries owing to its superior mechanical strength and corrosion resistance. However, its poor machinability, characterized by excessive tool wear, poor surface integrity, and high energy consumption, poses significant manufacturing challenges. To address these concerns, the influence of different lubrication and cooling strategies — Dry, MQL, Cryo-LN₂, and Cryo-CO₂— was systematically examined in terms of machining performance, energy efficiency, and economic viability. Based on the experimental findings, the following key conclusions were drawn:

- Tool wear was significantly minimized with Cryo-LN₂ achieving a reduction of 21.11%, followed by Cryo-CO₂ with 20%, and MQL with 16.22%, compared to dry machining conditions.
- Adhesion and abrasion were identified as the predominant tool wear mechanisms during machining of Hastelloy X. Adhesive wear caused built-up edge formation, while abrasive wear induced grooves on the tool surface. Cryogenic cooling effectively mitigated these wear mechanisms by reducing cutting zone temperature and friction.
- Surface finish was considerably improved with average surface roughness reductions of 25% using Cryo-LN₂, 22.22% with Cryo-CO₂, and 15.28% under MQL, as compared to dry cutting conditions.
- Cryo-CO₂ cooling demonstrated superior surface integrity by completely eliminating chip weldments from the machined surface, thus ensuring enhanced surface quality of the finished product.
- Energy consumption during machining was substantially reduced by 15.93% under Cryo-LN₂, 14.82% under Cryo-CO₂, and 7.30% under MQL environments relative to dry machining.
- The overall machining cost per component was minimized with Cryo-LN₂ delivering the highest cost savings of 26.67% over dry machining, followed by 7.37% and 1.12% cost benefits over MQL and Cryo-CO₂, respectively.
- Lastly, the Pugh matrix shows that cryogenic cooling methods are the most sustainable for machining Hastelloy X. They provide strong environmental and health advantages, along with better machining performance such as lower tool wear, improved surface finish, and reduced cutting temperatures.

## Future scope of work

The findings of this research open several promising directions for future investigation:

- While cryogenic LN₂ proved most effective in improving tool life and reducing machining costs, combining it with advanced lubricants like nano-enhanced MQL (e.g., graphene or MoS₂-based fluids) may offer even greater benefits. Future studies should explore hybrid systems like Cryo-MQL to assess their synergy on thermal control, tool wear, and surface finish.
- Integrating artificial intelligence and machine learning can help optimize cutting parameters in real-time based on tool condition, temperature, or surface quality. Predictive models could also enhance sustainability by minimizing energy use and extending tool life.
- The methods studied here should be applied to other nickel-based superalloys (e.g., Inconel 718, Hastelloy C276) and difficult-to-machine materials like titanium or cobalt-chrome alloys to confirm broader applicability.
- Future work should include full lifecycle analyses (LCA) and carbon footprint evaluations of the machining processes to understand long-term environmental impacts beyond immediate energy savings or cost reductions.
- Investigating how different tool coatings (e.g., diamond-like carbon or nano-layered coatings) and geometries perform under sustainable lubri-cooling could reveal further gains in efficiency and surface quality.
- Conducting long-term, shop-floor trials under actual production settings would validate lab findings and help develop practical guidelines for sustainable manufacturing in aerospace, biomedical, and chemical industries.

By pursuing these directions, future research can further strengthen the foundation for cleaner, more cost-effective, and efficient machining of superalloys.

## Data Availability

“The datasets used and/or analyzed during the current study will be available from the corresponding author upon reasonable request.”

## References

[1] Kurniawan, R. et al. Machinability of modified inconel 713 C using a WC TiAlN-coated tool. *J. Manuf. Process.* **57**, 409–430 (2020).

[2] Mallick, R., Kumar, R., Panda, A. & Sahoo, A. K. Current status of hard turning in manufacturing: aspects of cooling strategy and sustainability. *Lubricants* **11** (3), 108 (2023).

[3] Manikandan, K., Ranjith kumar, P., Raj kumar, D. & Palanikumar, K. Machinability evaluation and comparison of incoloy 825, inconel 603 XL, Monel K400 and inconel 600 super alloys in wire electrical discharge machining. *J. Mater. Res. Technol.* **9**, 12260–12272 (2020).

[4] Dutta, S. et al. A State-of-the-Art review on Micro-Machining of nitinol shape memory alloys and optimization of process variables considering the future trends of research. *J. Manuf. Mater. Process.* **9** (6), 183 (2025).

[5] Bhowmik, A. et al. A comprehensive review on the viability of minimum quantity lubrication technology for machining difficult-to-cut alloys. *AIP Adv.*, **15**(3). (2025).

[6] Bhowmik, A. et al. Advancing sustainability in EDM: A brief review of eco-friendly dielectric fluids. *AIP Adv.*, **15**(4). (2025).

[7] Sen, B. & Bhowmik, A. *Application of Minimum Quantity GnP Nanofluid and Cryogenic LN2 in the Machining of Hastelloy C276*194p.109509 (Tribology International, 2024).

[8] Sen, B., Debnath, S. & Bhowmik, A. Sustainable machining of Superalloy in minimum quantity lubrication environment: leveraging GEP-PSO hybrid optimization algorithm. *Int. J. Adv. Manuf. Technol.* **130** (9), 4575–4601 (2024).

[9] Peng, R., He, X., Tong, J., Tang, X. & Wu, Y. Application of a tailored eco-friendly nanofluid in pressurized internal-cooling grinding of inconel 718. *J. Clean. Prod.* **278**, 123498 (2021).

[10] Vukelic, D. et al. Evaluation of an environment-friendly turning process of inconel 601 in dry conditions. *J. Clean. Prod.* **266**, 121919 (2020).

[11] Benedicto, E., Carou, D. & Rubio, E. M. Technical, economic and environmental review of the lubrication/cooling systems used in machining processes. *Procedia Eng.* **184**, 99–116 (2017).

[12] Krolczyk, G. M. et al. Ecological trends in machining as a key factor in sustainable production – A review. *J. Clean. Prod.* **218**, 601–615 (2019).

[13] Devillez, A., Schneider, F., Dominiak, S., Dudzinski, D. & Larrouquere, D. Cutting forces and wear in dry machining of inconel 718 with coated carbide tools. *Wear* **262** (7–8), 931–942 (2007).

[14] Zhang, S., Li, J. F. & Wang, Y. W. Tool life and cutting forces in end milling inconel 718 under dry and minimum quantity cooling lubrication cutting conditions. *J. Clean. Prod.* **32**, 81–87 (2012).

[15] Tamang, S. K., Chandrasekaran, M. & Sahoo, A. K. Sustainable machining: an experimental investigation and optimization of machining inconel 825 with dry and MQL approach. *J. Brazilian Soc. Mech. Sci. Eng.* **40**, 1–18 (2018).

[16] Brito, P., Ramos, P. A., Resende, L. P., de Faria, D. A. & Ribas, O. K. Experimental investigation of cooling behavior and residual stresses for quenching with vegetable oils at different bath temperatures. *J. Clean. Prod.* **216**, 230–238 (2019).

[17] Li, M. et al. Parameter optimization during minimum quantity lubrication milling of TC4 alloy with graphene-dispersed vegetable-oil-based cutting fluid. *J. Clean. Prod.* **209**, 1508–1522 (2019).

[18] Prasad, M. M. S. Performance evaluation of nano graphite inclusions in cutting fluids with Mql technique in turning of Aisi 1040. *IJRET: Int. J. Res. Eng. Technol.* **2**, 381–393 (2013).

[19] Naresh Babu, M., Anandan, V., Muthukrishnan, N., Arivalagar, A. A. & Dinesh Babu, M. Evaluation of graphene based nano fluids with minimum quantity lubrication in turning of AISI D3 steel. *SN Appl. Sci.* **1**, 1–15 (2019).

[20] Danish, M., Gupta, M. K., Rubaiee, S., Ahmed, A. & Sarikaya, M. Influence of graphene reinforced sunflower oil on thermo-physical, tribological and machining characteristics of inconel 718. *J. Mater. Res. Technol.* **15**, 135–150 (2021).

[21] Grguraš, D., Sterle, L. & Pušavec, F. Cutting forces and chip morphology in LCO2 + MQL assisted robotic drilling of Ti6Al4V. *Procedia CIRP*. **102**, 299–302 (2021).

[22] Shokrani, A., Dhokia, V. & Newman, S. T. Investigation of the effects of cryogenic machining on surface integrity in CNC end milling of Ti–6Al–4V titanium alloy. *J. Manuf. Process.* **21**, 172–179 (2016).

[23] Shokrani, A., Al-Samarrai, I. & Newman, S. T. Hybrid cryogenic MQL for improving tool life in machining of Ti-6Al-4V titanium alloy. *J. Manuf. Process.* **43**, 229–243 (2019).

[24] Arafat, R., Madanchi, N., Thiede, S., Herrmann, C. & Skerlos, S. J. Supercritical carbon dioxide and minimum quantity lubrication in pendular surface grinding–A feasibility study. *J. Clean. Prod.* **296**, 126560 (2021).

[25] Khanna, N., Kshitij, G., Kashyap, N., Rashid, R. A. R. & Palanisamy, S. Machinability analysis for drilling Ti6Al4V ELI under sustainable techniques: EMQL vs LCO2. *Tribol. Int.* **188**, 108880 (2023).

[26] Agrawal, C. et al. Comprehensive analysis of tool wear, tool life, surface roughness, costing and carbon emissions in turning Ti–6Al–4V titanium alloy: cryogenic versus wet machining. *Tribol. Int.* **153**, 106597 (2021).

[27] Khanna, N., Agrawal, C., Gupta, M. K. & Song, Q. Tool wear and hole quality evaluation in cryogenic drilling of inconel 718 Superalloy. *Tribol. Int.* **143**, 106084 (2020).

[28] Pereira, O. et al. CO2 cryogenic milling of inconel 718: cutting forces and tool wear. *J. Mater. Res. Technol.* **9** (4), 8459–8468 (2020).

[29] Khanna, N. et al. Comparison of sustainable cooling/lubrication strategies for drilling of wire Arc additively manufactured inconel 625. *Tribol. Int.* **200**, 110068 (2024).

[30] Sen, B., Hussain, S. A. I., Mia, M., Mandal, U. K. & Mondal, S. P. Selection of an ideal MQL-assisted milling condition: an NSGA-II-coupled TOPSIS approach for improving machinability of inconel 690. *Int. J. Adv. Manuf. Technol.* **103**, 1811–1829 (2019).

[31] Sen, B. et al. Comparative analysis of NSGA-II and TLBO for optimizing machining parameters of inconel 690: A sustainable manufacturing paradigm. *J. Mater. Eng. Perform.*, 1–16. (2024).

[32] Gupta, M. K. et al. Environment and economic burden of sustainable cooling/lubrication methods in machining of Inconel-800. *J. Clean. Prod.* **287**, 125074 (2021).

[33] Sen, B. et al. Minimum quantity blended bio-lubricants for sustainable machining of superalloy: an MCDM model-based study. *AIP Adv.*, **14**(7). (2024).

[34] Mallick, R., Kumar, R., Panda, A. & Sahoo, A. K. Hard turning performance evaluation in various sustainable environments employing a COPRAS optimization and Pugh matrix sustainability approach. *Arab. J. Sci. Eng.*, 1–39. (2024).

